# Part-time versus full-time employment and mental health for people with and without disability

**DOI:** 10.1016/j.ssmph.2023.101446

**Published:** 2023-06-07

**Authors:** Lu Ye, Anne Kavanagh, Dennis Petrie, Helen Dickinson, Zoe Aitken

**Affiliations:** a Disability and Health Unit, Centre for Health Equity, Melbourne School of Population and Global Health, The University of Melbourne, Parkville, Victoria, Australia; b Centre for Health Economics, Monash Business School, Monash University, Caulfield East, Victoria, Australia; c Public Service Research Group, School of Business, UNSW Canberra, Canberra, ACT, Australia

**Keywords:** Health inequalities, Mental health, Disability, Part-time and full-time employment, Fixed-effects analysis, Effect modification

## Abstract

**Objectives:**

This paper investigates the relationship between part-time and full-time employment and mental health for people with and without disability, as well as differences in the relationship by age and sex.

**Methods:**

Using data from 13,219 working-aged people (15–64 years) in the labour force who participated in five annual waves of a longitudinal cohort study in Australia, the analysis used fixed effect regression models to examine within-person changes in mental health associated with changes in employment status (full-time; part-time; unemployed). Differences in the relationship between employment status and mental health by disability, sex, and age were assessed.

**Results:**

Among people with disability, there was evidence that working part-time and full-time were associated with a 4.2-point (95% CI 2.6, 5.7) and 6.0-point (95% CI 4.4, 7.6) increase in mental health scores compared with when they were unemployed. For people without disability, there were much smaller differences in mental health associated with working part-time (***β*** = 1.0, 95% CI 0.2, 1.9) and full-time (***β*** = 1.4, 95% CI 0.5, 2.2) compared with when they were unemployed. The positive effects of both part-time and full-time employment were of greater magnitude for people with disability aged younger than 45 years compared to those aged 45 years and older.

**Conclusions:**

The results of this study suggest that both part-time and full-time employment may have beneficial effects on the mental health of people with disability, particularly for younger people. The findings underscore the value of employment for people with disability, given we found much larger beneficial mental health effects in comparison to people without disability.

## Highlights

• Part-time and full-time employment have beneficial effects on mental health.
• The beneficial effects were larger for people with disability compared to those without disability.
• Government policies should recognise the value of part-time employment for people with disability.
• Government policies need to address employment barriers to close the gap in employment inequalities.

## 1 Introduction

In 2021, there are approximately 16% of the world's population live with a disability and an estimation of 785 million people with disability are of working-age (15–64 years) (International Labour Organization, 2017; World Health Organization, 2022). People with disability are more likely to have poorer health outcomes, including higher risks of mental health issues such as depression or anxiety, than those without disability (World Health Organization, 2022). People with disability also experience significant employment inequalities compared with those without. In many countries, people with disability are at least twice as likely to be unemployed, less likely to be employed (36% versus 60%, on average) and more likely to work part-time compared to their non-disabled peers (Department of Economic and Social Affairs, 2007; Department of Economic and Social Affairs, 2018). People with psychological disability are found least likely to be employed compared to those with other types of disability (Boman et al., 2015). In addition, age and sex inequalities have been found in most countries that, among people with disability, older people and women are less likely to be employed than younger groups and men (Department of Economic and Social Affairs, 2007; Department of Economic and Social Affairs, 2018). Age and sex heterogeneity has also been found between studies in a recent review focusing on the effect of unemployment on health outcomes in different subgroups (Norström et al., 2014).

Jahoda developed the latent deprivation model that proposed that unemployment was linked to the deprivation of psychological wellbeing through five latent consequences including time structure, social contacts, social purposes, identity and activity (Jahoda, 1981). Empirical research provides evidence supporting this theory, demonstrating that unemployment is associated with decreases in perceived competence, activity, life satisfaction, self-esteem, and vigour, and with increases in depression, psychological distress, tension, fatigue, and confusion (Feather & O'Brien, 1986; Muller et al., 1993). There is also increasing evidence that disability-related mental health inequalities are in part driven by socio-economic factors with unemployment identified as a key factor involved in the association (Aitken et al., 2018, 2021). The beneficial effects of employment on mental health have been stated in many studies, including reduce the risk of depression and anxiety, and increase autonomy, sense of value, status, social engagement, personal satisfaction, confidence, and accelerating the recovery from mental illness (Boardman et al., 1999; Claussen et al., 1993; Modini et al., 2016). As this association are found to be related to household income and social support (Van Aerden et al., 2017), the positive relationship between employment and mental health may be stronger for people with disability (Saunders & Nedelec, 2014) because of their lower income and limited social networks (World Health Organization, 2022).

Although employment is often seen as a positive activity, it does not always benefit people's health, including those with a disability. Being employed in low-quality jobs with low earnings, few benefits, little training, poor flexibility, and a high level of instability has been found to have an impact on mental health similar to that of unemployment (Van Aerden et al., 2017). Researchers have highlighted the need for people with disability and mental illness to find jobs that suit them, balancing challenge, flexibility and predictability (Saunders & Nedelec, 2014). However, there is no research of how much employment (part-time vs full-time) is needed to receive mental health benefits and also whether the quantity needed differs for those with and without disability.

Many researchers have pointed out that part-time employment may be a stepping-stone for people with disability to move from unemployment to employment (Jones, 2007; Pagán, 2007). People with disability may not have the time and energy to devote to work full-time because of their functional limitations (Centers for Disease Control and Prevention, 2022), and self-care needs (health care, transportation, assistance, or rehabilitation) (Pagan-Rodriguez, 2009). Thus, part-time employment could be an effective work arrangement for people with disability that provides flexible working conditions to suit their needs. Other studies have noted that people with disability work part-time due to their inability to find full-time jobs because of workplace discrimination and stigma (Pagán, 2007; Schur, 2003). And involvement in part-time work due to an inability to find full-time work has been found to be associated with poor mental health (Van Aerden et al., 2017). As such, part-time employment may potentially be beneficial or detrimental to the mental health of people with disability compared to full-time employment, but there is no evidence to support this.

This study explores the impact of disability on the association between employment status and mental health, examining the effect of part-time and full-time employment in comparison to unemployment on mental health for people with and without disability. This study further investigates the result by estimating the effects in different subgroups, such as age and sex. In this context, this study uses data from working aged Australians (aged between 15 and 64 years old) to advance understanding of the link between part-time employment and mental health, how the effect compares between people with and without disability, and whether differential effects exist by age group and sex.

## 2 Material and methods

### 2.1 Data source

The data used in this study was obtained from the Household, Income and Labour Dynamics in Australia (HILDA) Survey, a nationally representative longitudinal study of Australian households conducted annually since 2001 (Department of Social Services, 2019). The survey uses a combination of interviews and self-reported questionnaires to gather information annually from 17,462 individuals from 9664 households in wave 19 after topping up (Melbourne Institute, 2020). On average, for all waves of the survey, response rates of the in-scope participants were approximately 70% (ranging from 60% to 81%), and attrition was 5.7% between waves, ranging from 3.5% in 2014 to 13.2% in 2002 (Aitken et al., 2018; Department of Social Service, 2022a, Department of Social Services, 2022b).

The analysis for this study included data from 5 annual waves of HILDA (2015–2019), with the population of interest restricted to working aged (15–64 years) participants who were in the labour force.

### 2.2 Outcome variable

Mental health was evaluated using the Five-Item Mental Health Inventory (MHI-5), a subscale of the Short Form 36 health questionnaire, which is an effective instrument for investigating depression, anxiety, and panic symptoms, given its high sensitivity and specificity for detecting depression, anxiety, and panic disorders among the general population (Yamazaki et al., 2005). It assesses five items relating to mental health, including nervousness, mood and feeling down and feeling calm and happy over the past 4 weeks, using the five response categories for each score (Yamazaki et al., 2005). Total raw scores are transformed into a continuous scale ranging from 0 to 100, with higher scores indicating better mental health. The mean score on the MHI-5 in HILDA was approximately 73.60, with a standard deviation of 17.47. A difference in the MHI-5 score of 4–5 points reflects a clinically significant difference in mental health (Knox & King, 2009).

### 2.3 Exposure variable

Employment status was categorised as part-time employment, full-time employment, and unemployment, with people who were not in the labour force (those who were neither employed nor unemployed during the survey reference week (Australian Bureau of Statistics, 2001)) excluded from the analysis. Part-time employment was defined as usually working less than 35 h per week (Australian Bureau of Statistics, 2018). Full-time employment was defined as usually working 35 h or more per week (Australian Bureau of Statistics, 2019). Participants were categorised as unemployed if they reported not being in paid employment but were actively looking for jobs and available to work (Australian Bureau of Statistics, 2019).

### 2.4 Potential confounders

**Fig. 1:**
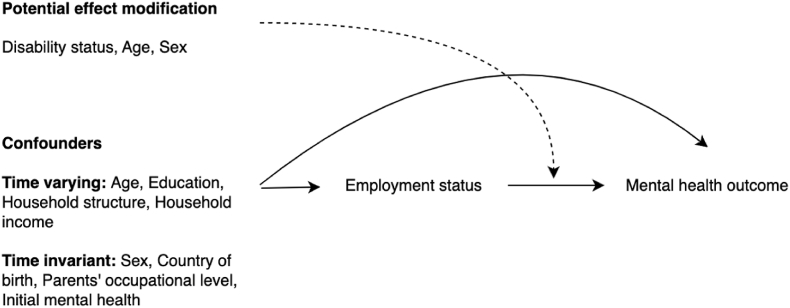
Directed Acyclic Graph illustrating causal relationships between employment status and mental health outcomeand potential confounders and effect modifiers.

Factors that could be common causes of employment status (unemployment, part-time employment, and full-time employment) and mental health were chosen to be potential confounders, selected using the directed acyclic graph (see Fig. 1). Confounders included age (15–24, 25–34, 35–44, 45–54, 55–64 years), sex (female, male), country of birth (Australia, other English speaking, non-English speaking country), education (bachelor's degree or higher, advanced diploma or certificate, year 12, less than year 12), parents' occupational skill level (never worked, low skill, medium skill, high skill), household structure (couple no children, couple with children, single no children, single with children), and initial mental health.

Household income was considered but not included as a potential confounder as it could be an important mediator on the causal pathway from employment status to mental health.

### 2.5 Effect modifiers

Firstly, disability status (with disability, without disability) was assessed as an effect modifier in this study, to assess whether there were differences in the effect of employment status on mental health for people with and without disability. Disability status was asked in a single question in the HILDA Survey, asking whether people had “any limitation, long-term health condition or impairment that restricts everyday activities lasting at least six months” (Australian Bureau of Statistics, 2019). If answer “yes”, they were then asked questions about the type of condition, using a showcard listing specific examples such as long term sight, hearing and speech problems; limited use of arms or fingers and feet or legs; difficulty learning or understanding things; or a nervous or emotional condition which requires treatment or any mental illness which requires help or supervision. The responses were used to identify people who reported a psychological disability, defined as those reporting a nervous or emotional condition which requires treatment or any mental illness which requires help or supervision, and those who reported non-psychological disability (Aitken et al., 2017). Considering disability status could change over time, we created a time-invariant indicator of disability status that identified if an individual reported a disability in at least 60% of waves contributing to the analysis (the ‘disability’ group) (Kariuki et al., 2011). Participants with disability were compared with those who reported a disability in less than 20% of waves (the ‘no disability’ group). People who reported other patterns of disability status were excluded from the sample. As such, the disability indicator did not vary within an individual over time.

Secondly, age group and sex were examined as effect modifiers in this study to examine differences in the effect of part-time employment on mental health according to categories of age and sex.

### 2.6 Fixed effects models

The directed acyclic graph illustrates many common causes of employment status and mental health, leading to potential confounding from both time-varying and time-invariant confounders. Since between-person differences in characteristics (e.g., sex, ethnicity) of people in the exposure groups (e.g., employed full-time versus unemployed) may bias the estimate of the causal effect of employment status on mental health, analytic strategies that estimate the association between changes in exposures and outcomes within-individuals can be used to address the problem of confounding and maximize causal inference (Gunasekara et al., 2014).

Fixed-effects regression models is an effective way to analyse longitudinal data by modelling the within-person variation in exposures and outcomes to adjust for both observed and unobserved time-invariant confounders (Gunasekara et al., 2014). In fixed-effects models, each person serves as their own control (Aitken, 2019, p. 63). The relationship between changes in exposures and changes in outcomes over time can be achieved by repeated measures of longitudinal data on the same individuals (Aitken, 2019, p. 63). The models generate an estimate of the individual effect of the exposure on the outcome by comparing the outcome when each person was ‘exposed’ with when they were ‘unexposed’. The individual effects are then averaged to generate an average causal effect across the whole sample.

In this study, fixed-effects regression models were used to estimate the association between the MHI-5 score and employment status. Coefficients generated from these models describe differences in the MHI-5 score associated with being employed part-time and full-time compared with that individual's mean MHI-5 score when they were unemployed.

### 2.7 Statistical analysis

All statistical analyses were performed using Stata/SE 17.0. First, descriptive analyses were performed to describe the characteristics of the population of interest and the distribution of the MHI-5 score, by disability status and by employment status in the first wave contributing to the analysis.

Second, we used a fixed-effects longitudinal regression model to examine the association between employment status and mental health in working-aged Australians. Since this is a within-person analysis, time-invariant confounding factors, including sex, country of birth, and parents’ occupational skill level, prior mental health, were implicitly adjusted for in the fixed-effects analysis, as they did not vary over time within individuals. Time-varying confounding factors, including age group, education (which may vary over time particularly for the 15–24-year-old group), and household structure, were adjusted for in the analysis.

To assess the differences in the relationship between employment status and mental health according to disability status, we conducted a test for interaction using a likelihood ratio test (LRT) to assess whether the effect of employment status on mental health was different for people with and without disability. There was strong evidence of interaction (p < 0.001), therefore an interaction term between disability and employment status was included in all models and results were presented separately for people with and without disability.

We also tested for interactions between both age group and sex with employment status. There was no evidence of an interaction with sex (p = 0.747). There was evidence of an interaction with age group (p = 0.004), and we further tested the fit of a three-way interaction between employment status, disability and age group compared to models with two two-way interactions and found evidence supporting the use of the three-way interaction in the models (p = 0.0002). Therefore, to examine differences by age group, we included a three-way interaction term between employment status, disability, and age group and presented the results separately by disability status and age group.

Finally, a sensitivity analysis was used to test the robustness of the findings. In this analysis, people with psychological disability (those with nervous or emotional conditions that require treatment and those with mental illness that require help or supervision) were excluded from the sample since they are likely to have poorer mental health associated with disability and are the most disadvantaged group in the labour market compared to other types of disability (Boman et al., 2015). The fixed effects models were run on this reduced sample to estimate the effect of employment status on mental health for people with disability other than a psychological disability.

## 3 Results

**Fig. 2:**
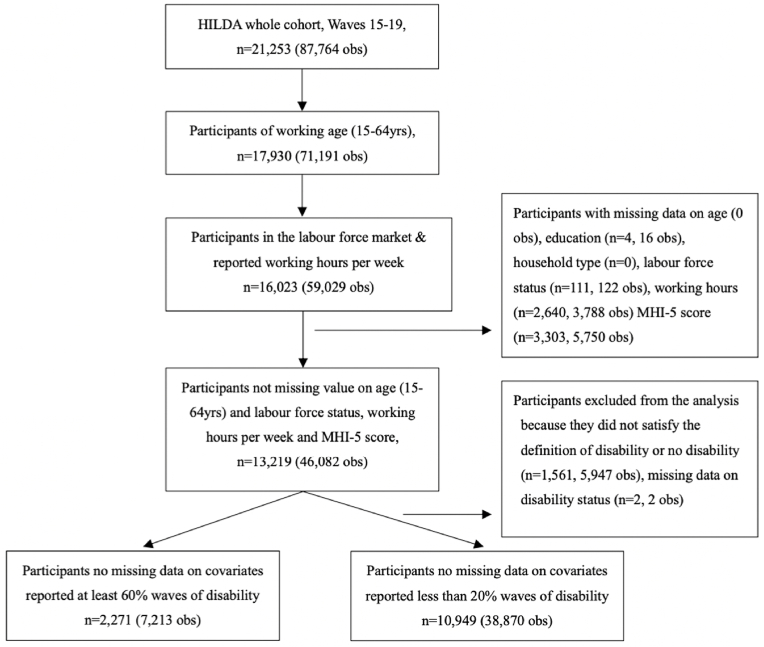
Flow chart of selecting study sample, HILDA, 2015 to 2019.

The analytic sample across all contributing waves amounted to 13,219 participants (46,082 observations). Of these, 10,949 participants (38,870 observations) reported not having disability, and 2271 participants (7213 observations) reported having disability. The selection of participants in the analysis sample is shown in Fig. 2.

Table 1 presents the sample characteristics according to disability status in each person's first wave contributing to the analysis. In our sample consisting of people of working age who were in the labour force, people with disability were on average older than people without disability. People with disability were less likely to work full-time compared to people without disability (45.3% versus 57.1%), more likely to work part-time (36.8% versus 33.7%), and more likely to be unemployed (17.9% versus 9.2%). People with disability had a lower mean MHI-5 score than those without disability (65.6 versus 75.0). For people with and without disability, the MHI-5 score increased with working hours, with lowest MHI-5 score for people who were unemployed and highest MHI-5 scores for those employed full-time. On average, mental health scores were lower for people with disability compared to those without, for all categories of employment status (Table 2). A descriptive analysis comparing sample characteristics by employment status can be seen in Supplementary Table 1.

**Table 1:** Sample characteristics measured at each person's first wave of data, by disability status.

	No disability n = 10,039 (%)	Has disability n = 2114 (%)
**Age (%)**		
15–24 years	2867 (28.6)	450 (21.3)
25–34 years	2549 (25.4)	380 (18.0)
35–44 years	1806 (18.0)	338 (16.0)
45–54 years	1784 (17.8)	494 (23.4)
55–64 years	1031 (10.3)	451 (21.3)
**Sex (%)**		
Male	4992 (49.7)	1016 (48.1)
Female	5045 (50.3)	1097 (51.9)
**Country of birth (%)**		
Australia	8143 (81.1)	1799 (85.1)
Other English speaking	770 (7.7)	166 (7.9)
Non-English speaking	1124 (11.2)	148 (7.0)
**Household structure (%)**		
Couple no children	2520 (26.9)	591 (30.7)
Couple with children	4884 (52.2)	814 (42.3)
Single no children	1024 (10.9)	264 (13.7)
Single with children	928 (9.9)	257 (13.3)
**Employment status (%)**		
Unemployed	918 (9.2)	378 (17.9)
Employed part-time	3385 (33.7)	778 (36.8)
Employed full-time	5734 (57.1)	957 (45.3)
**Education (%)**		
Bachelor's degree or higher	3046 (30.4)	456 (21.6)
Diploma or certificate	3053 (30.4)	799 (37.8)
Secondary education	1879 (18.7)	315 (14.9)
Less than secondary education	2059 (20.5)	543 (25.7)
**Parents occupation (%)**		
Never worked or low skill	1348 (13.7)	395 (19.0)
Medium skill	3298 (33.5)	769 (37.0)
High skill	5207 (52.9)	913 (44.0)
MHI-5 (mean ± SD)	75.0 ± 15.6	65.6 ± 19.9

**Table 2:** Mental health scores measured at each person's first wave of data, by disability and employment status.

	No disability, mean (SD)	Disability, mean (SD)
Unemployed	68.1 (8.6)	57.9 (21.1)
Employed PT	74.9 (15.7)	65.7 (19.9)
Employed FT	76.3 (14.9)	68.2 (19.3)

The results of the fixed-effect analysis (Table 3) showed that, for people with disability, MHI-5 scores were on average 4.2 points higher when they worked part-time (95% CI 2.6 to 5.7) and 6.0 points higher when they worked full-time (95% CI 4.4 to 7.6) compared to when they were unemployed. Among people without disability, part-time employment and full-time employment was associated with a 1.0-point higher score (95% CI 0.2 to 1.9) and a 1.4-point higher score (95% CI 0.5 to 2.2) on the MHI-5 scale compared with when they were unemployed, respectively.

**Table 3:** Regression results from the fixed-effects model representing estimated mean differences in MHI-5 scores associated with changing employment status relative to unemployment, stratified by disability status.

	No disability	Disability	Measure of effect modification on additive scale
	Coef. (95% CI; p value)	Coef. (95% CI; p value)	Coef. (95% CI; p value)
**Employment status**			
Unemployed	0	0	0
Employed PT	1.0 (0.2, 1.9; p = 0.016)	4.2 (2.6, 5.7; p < 0.001)	3.1 (1.4, 4.9; p < 0.001)
Employed FT	1.4 (0.5, 2.2; p = 0.002)	6.0 (4.4, 7.6; p < 0.001)	4.6 (2.8, 6.5; p < 0.001)

* Adjusted for: age, education, household type.

^Coefficients refer to estimated mean difference in the MHI-5 score.

The results disaggregated by age group provide evidence that younger people with disability (aged less than 45 years) experienced larger clinically significant beneficial effects of both part-time and full-time employment on mental health compared to people with disability aged over 45 years (see Table 4, column 3), noting that a 4- to 5- point difference in MHI score is believed to represent a clinically important difference (Knox & King, 2009). Among people with disability aged 15–24 years, part-time and full-time employment was associated with average increases in MHI-5 scores of 5.8-points (95% CI 3.0, 8.6) and 4.8-points (95% CI 1.6, 8.0) respectively, compared to when they were unemployed. Among people aged 25–34 years, part-time and full-time employment were associated with 3.6-point (95% CI 0.3 to 7.0) and 7.9-point (95% CI 4.4 to 11.3) increases on the MHI-5 scale compared to unemployment. Among people aged 35–44 years, working part-time and full-time was associated with 7.1-point (95% CI 3.1 to 11.2) and 8.7-point (95% CI 4.6 to 12.9) increases on the MHI-5 scale compared to when they were unemployed. For people aged 45–54 years old, full-time employment was associated with a 5.1-point increase in MHI-5 score (95% CI 1.7 to 8.6) compared to unemployment and there was no evidence of an effect of part-time employment on mental health (estimated mean difference = 1.5, 95% CI -1.9 to 5.0). For people aged 55–64 years, there was no evidence of effects of part-time and full-time employment on mental health.

**Table 4:** Regression results from the fixed-effects model representing estimated mean differences in MHI-5 scores associated with changing employment status relative to unemployment, stratified by disability status and age group.

	No disability	Disability	Measure of effect modification by disability on additive scale
	Coef. (95% CI; p value)	Coef. (95% CI; p value)	Coef. (95% CI; p value)
**15**–**24 yrs**			
**Unemployed**	0	0	0
**Employed PT**	−0.5 (−1.7, 0.7; p = 0.388)	5.8 (3.0, 8.6; p < 0.001)	6.3 (3.3, 9.3; p < 0.001)
**Employed FT**	−0.3 (−1.6, 1.0; p = 0.690)	4.8 (1.6, 8.0; p = 0.003)	5.0 (1.6, 8.4; p = 0.004)
**25**–**34 yrs**			
**Unemployed**	0	0	0
**Employed PT**	2.6 (0.7, 4.6; p = 0.008)	3.6 (0.3, 7.0; p = 0.033)	1.0 (−2.9, 4.9; p = 0.614)
**Employed FT**	2.9 (1.0, 4.8; p = 0.003)	7.9 (4.4, 11.3; p < 0.001)	5.0 (1.0, 8.9; p = 0.013)
**35**–**44 yrs**			
**Unemployed**	0	0	0
**Employed PT**	1.3 (−0.9, 3.4; p = 0.250)	7.1 (3.1, 11.2, p = 0.001)	5.9 (1.3, 10.5; p = 0.012)
**Employed FT**	1.3 (−0.8, 3.4; p = 0.236)	8.7 (4.6, 12.9; p < 0.001)	7.4 (2.8, 12.1; p = 0.002)
**45**–**54 yrs**			
**Unemployed**	0	0	0
**Employed PT**	4.1 (1.8, 6.4; p < 0.001)	1.5 (−1.9, 5.0; p = 0.381)	−2.6 (−6.7, 1.6; p = 0.227)
**Employed FT**	4.6 (2.3, 6.8; p < 0.001)	5.1 (1.7, 8.6; p = 0.004)	0.5 (−3.6, 4.6; p = 0.800)
**55**–**64 yrs**			
**Unemployed**	0	0	0
**Employed PT**	0.9 (−2.5, 4.4; p = 0.587)	0.7 (−3.8, 5.2; p = 0.764)	−0.3 (−5.9, 5.4; p = 0.929)
**Employed FT**	0.9 (−2.5, 4.3; p = 0.596)	1.3 (−3.2, 5.9; p = 0.570)	0.4 (−5.3, 6.1; p = 0.889)
**Measure of effect modification by age group on additive scale**			3-way interaction terms*
**25**–**34 yrs**			
**Employed PT**	3.2 (0.9, 5.5; p = 0.007)	−2.2 (−6.5, 2.2; p = 0.327)	−5.3 (−10.2, −0.4; p = 0.033)
**Employed FT**	3.2 (0.9, 5.5; p = 0.007)	3.1 (−1.6, 7.8; p = 0.192)	−0.1 (−5.3, 5.1; p = 0.980)
**35**–**44 yrs**			
**Employed PT**	1.8 (−0.7, 4.3; p = 0.154)	1.3 (−3.6, 6.2; p = 0.594)	−0.5 (−6.0, 5.0; p = 0.867)
**Employed FT**	1.5 (−0.9, 4.0; p = 0.223)	3.9 (−1.3, 9.2; p = 0.139)	2.4 (−3.4, 8.2; p = 0.414)
**45**–**54 yrs**			
**Employed PT**	4.6 (2.0, 7.2; p < 0.001)	−4.3 (−8.7, 0.1; p = 0.058)	−8.9 (−14.0, −3.8; p = 0.001)
**Employed FT**	4.8 (2.3, 7.4; p < 0.001)	0.3 (−4.4, 5.0; p = 0.885)	−4.5 (−9.8, 0.9; p = 0.100)
**55**–**64 yrs**			
**Employed PT**	1.5 (−2.1, 5.1; p = 0.425)	−5.1 (−10.4, 0.2; p = 0.058)	−6.6 (−13.0, −0.2, p = 0.044)
**Employed FT**	1.2 (−2.4, 4.8; p = 0.523)	−3.4 (−9.0, 2.1; p = 0.224)	−4.6 (−11.2, 2.0; p = 0.171)

* 3-way interaction term describing the additional effect of PT and FT employment on mental health for people with disability for each of the age groups.

* Adjusted for: age, education, household type.

^Coefficients refer to estimated mean difference in the MHI-5 score.

Among people without disability, there was no evidence of an effect on mental health for part-time or full-time work for those aged 15–24 years, 35–44 years, and 55–64 years (Table 4, column 2). Working in part-time and full-time jobs for people aged 25–34 years was associated with an increase of 2.6 points (95% CI 0.7 to 4.6) and 2.9 points (95% CI 1.0 to 4.8) on the MHI-5 scale compared to when they were unemployed. For people aged 45–54 years, working part-time and full-time was associated with an increase of 4.1 points (95% CI 1.8 to 6.4) and 4.6 points (95% CI 2.3 to 6.8) on the MHI-5 scale compared to when they were unemployed.

The measures of effect modification by disability (Table 4, column 4) estimate the additional mean difference in MHI-5 score between categories of employment status experienced by people with disability. For example, for people aged 15–24 years, the difference in mean MHI-5 score between people who were employed part-time and those unemployed was 6.3-points higher for people with a disability compared to those without disability. The measures of effect modification by age group estimate the additional mean difference in MHI-5 score between categories of employment status experienced by people of different age groups. The 3-way interaction terms estimate the additional mean difference in MHI-5 scores between categories of employment status experienced by people with disability in each of the four age groups older than 24 years, thus assessing whether the joint effect of having a disability and being of older age on the mean difference in mental health score by employment status was greater (or less) than the sum of their individual effects.

### 3.1 Sensitivity analysis

When people with psychological disability were excluded from the analysis, the number of people with disability reduced from 2271 (7217 obs) to 1974 (5931 obs). This exclusion minimally changed the number of people without disability (who may have reported psychological disability in a single wave and thus been excluded), with 138 people (138 obs) excluded from the analysis.

Results of the fixed-effects analysis (Table 5) demonstrated a similar pattern but estimated mean differences in mental health were of smaller magnitude compared to the primary analysis. For people without disability, the results were largely unchanged from the primary analysis.

**Table 5:** Regression results from the sensitivity analysis excluding people with a psychological disability, representing estimated mean differences in MHI-5 scores associated with changing employment status relative to unemployment, stratified by disability status (above) and age and employment status (below).

	No disability	Disability	Measure of effect modification on additive scale
	Coef. (95% CI; p value)	Coef. (95% CI; p value)	Coef. (95% CI; p value)
**Whole sample**			
**Unemployed**	0	0	0
**Employed PT**	0.9 (0.1, 1.8; p = 0.023)	2.3 (0.3, 4.2; p = 0.021)	1.3 (−0.8, 3.4; p = 0.219)
**Employed FT**	1.3 (0.5, 2.1; p = 0.002)	4.7 (2.8, 6.6; p < 0.001)	3.4 (1.3, 5.5; p = 0.002)
**15**–**24 yrs**			
**Unemployed**	0	0	0
**Employed PT**	−0.6 (−1.8, 0.5; p = 0.280)	3.4 (−0.2, 6.9; p = 0.062)	4.0 (0.3, 7.7; p = 0.034)
**Employed FT**	−0.4 (−1.6, 0.9; p = 0.581)	5.5 (1.6, 9.3; p = 0.006)	5.8 (1.7, 9.9; p = 0.005)
**25**–**34 yrs**			
**Unemployed**	0	0	0
**Employed PT**	2.5 (0.6, 4.4; p = 0.011)	4.6 (0.2, 9.1; p = 0.042)	2.1 (−2.7, 7.0; p = 0.392)
**Employed FT**	2.8 (1.0, 4.7; p = 0.003)	7.8 (3.4, 12.3; p < 0.001)	5.0 (0.2, 9.8; p = 0.040)
**35**–**44 yrs**			
**Unemployed**	0	0	0
**Employed PT**	1.3 (−0.8, 3.5; p = 0.224)	4.1 (−1.1, 9.3; p = 0.119)	2.8 (−2.8, 8.4; p = 0.327)
**Employed FT**	1.3 (−0.8, 3.4; p = 0.215)	7.1 (1.8, 12.3; p = 0.009)	5.7 (0.1, 11.4; p = 0.047)
**45**–**54 yrs**			
**Unemployed**	0	0	0
**Employed PT**	4.2 (1.9, 6.5; p < 0.001)	−0.8 (−4.7, 3.2; p = 0.700)	−5.0 (−9.5, −0.4; p = 0.032)
**Employed FT**	4,7 (2.5, 6.9; p < 0.001)	2.7 (−1.3, 6.6; p = 0.185)	−2.1 (−6.6, 2.4; p = 0.370)
**55**–**64 yrs**			
**Unemployed**	0	0	0
**Employed PT**	0.9 (-2.4, 4.3; p = 0.581)	−0.6 (-5.7, 4.4; p = 0.804)	−1.6 (-7.6, 4.5; p = 0.609)
**Employed FT**	0.9 (-2.4, 4.2; p = 0.586)	−0.0 (-5.0, 5.0; p = 0.997)	−0.9 (-6.9, 5.1; p = 0.761)
**Measure of effect modification by age group**			3-way interaction terms*
**25**–**34 yrs**			
**Employed PT**	3.2 (0.9, 5.4; p = 0.006)	1.3 (−4.4, 6.9; p = 0.663)	−1.9 (−8.0, 4.2; p = 0.544)
**Employed FT**	3.2 (0.9, 5.5; p = 0.006)	2.4 (−3.5, 8.3; p = 0.424)	−0.8 (−7.1, 5.5; p = 0.804)
**35**–**44 yrs**			
**Employed PT**	2.0 (−0.5, 4.4; p = 0.112)	0.8 (−5.5, 7.1; p = 0.808)	−1.2 (−7.9, 5.5; p = 0,792)
**Employed FT**	1.7 (−0.8, 4.1; p = 0.178)	1.6 (−4.9, 8.2; p = 0.631)	−0.1 (−7.0, 6.9; p = 0.985)
**45**–**54 yrs**			
**Employed PT**	4.8 (2.3, 7.4; p < 0.001)	−4.1 (−9.4, 1.2; p = 0.126)	−9.0 (−14.9, −3.0; p = 0.003)
**Employed FT**	5.1 (2.5, 7.6; p < 0.001)	−2.8 (−8.3, 2.7; p = 0.321)	−7.9 (−13.9, −1.8; p = 0.011)
**55**–**64 yrs**			
**Employed PT**	1.6 (−2.0, 5.1; p = 0.380)	−4.0 (−10.1, 2.1; p = 0.202)	−5.6 (−12.7, 1.5; p = 0.123)
**Employed FT**	1.3 (−2.3, 4.8; p = 0.480)	−5.5 (−11.8, 0.9; p = 0.090)	−6.7 (−14.0, 0.5; p = 0.068)

* 3-way interaction term describing the additional effect of PT and FT employment on mental health for people with disability for each of the age groups.

* Adjusted for: age, education, household type.

^Coefficients refer to estimated mean difference in the MHI-5 trans score on a 0–100 scale.

## 4 Discussion

### 4.1 Interpretation of findings

The main findings suggested that part-time employment was associated with increases in mental health and that positive associations of both part-time and full-time employment are stronger for people with disability compared to those without disability. Among people with disability younger than 45 years, part-time and full-time were both associated with clinically meaningful improvements in mental health on a self-reported basis.

The beneficial effect of full-time and part-time employment on mental health compared with unemployment for people with disability was consistent with the findings by Milner et al. (Milner et al., 2014), which demonstrated that moving from employment to unemployment was associated with large negative effects on mental health for people with disability, and effects of greater magnitude compared to those without disability. However, the study by Milner et al. did not distinguish between the impact of part-time and full-time employment. Our study has added to the evidence base by separately examining the effects of part-time and full-time employment and found that the impact on mental health was similar in magnitude for part-time employment compared to full-time employment. Part-time employment may also best suit some people with disability as an important avenue to transition into the job market.

Although gender differences in disability, employment status, and mental health have been pointed out in previous literature, such that women are more likely to be exposed to unemployment (Naami, 2015), part-time employment (Jones et al., 2006), and poorer mental health (Miller & Dishon, 2006), this study did not find evidence of differential effects of employment status on mental health by sex. However, our study found strong interaction with age, with evidence of stronger effects of part-time and full-time employment on mental health for younger people compared to older people with disability. Our study demonstrated large mental health benefits associated with part-time and full-time employment for people with disability aged 15–44 years and large mental health benefits of full-time for people with disability aged 45–54 years. In contrast, we found no evidence of an association between part-time employment and mental health for people with disability aged 45–64 years. This is surprising because, for people without disability, the beneficial effect of part-time and full-time employment on mental health was greater for older people compared to younger people. It may be that older people with disability are more likely to be in lower quality part-time employment, with less beneficial mental health effects, or they may be more likely to be employed in jobs with poor psychosocial working conditions or discrimination (Bjelland et al., 2010). Alternatively, it could be that older Australians with disability have stronger networks and, as a result, do not demonstrate the same benefits associated with employment compared to younger people with disability, or that it is explained by a cohort effect relating to changes in the employment environment for people with disability over time.

The findings have important policy implications. The Australian Government has recently released a new disability employment strategy ‘Employ My Ability’, which serves as a guiding framework for governments, employers, and the broader community to increase employment outcomes for people with disability (Department of Social Service, 2022a, Department of Social Services, 2022b). However, the strategy concentrates predominantly on increasing employment outcomes for young people with disability and people with high job capacity, by providing employers with online resources and support, refining recruitment processes, and building networks with organizations (Kuhn et al., 2009). The strategy also encourages the income support, tax and the industrial relations systems to work together to achieve full-time employment outcomes for people with disability (Department of Social Service, 2022a, Department of Social Services, 2022b) without considering part-time employment as a beneficial employment outcome in itself to strive towards. The findings suggest that employment targets of government policies should not be limited to people with disability who have the job capacity to work full-time and should be tailored to the characteristics (and preference) of people with disability, to recognise the value of part-time employment for some people with disability, and to increase the prevalence of part-time employment among people with disability who are currently unemployed. Alongside this, strategies are needed that encourage employers to hire people with disability (including in part-time jobs), develop education programs to prevent workplace discrimination, and provide education and training opportunities for people with disability, to reduce employment barriers and address the inequalities in employment opportunities between people with and without disability.

### 4.2 Strengths and limitations

The strengths of the study relate to the data source and methods used to estimate the effect of changes in employment status on mental health. The HILDA survey is a large nationally representative survey that collects detailed information about adults annually and presented a large sample size to conduct our analysis. The longitudinal design using five waves of data enabled us to characterise changes in individuals’ employment status and mental health over time. In our sample, the demographic and socio-economic characteristics of people in full-time, part-time, and unemployed varied considerably, which could have led to residual confounding. As such, we used fixed-effects regression models which produce robust estimates of causal effects of exposures on outcomes for confounders that were time invariant.

There were also limitations that needed to be taken into consideration. Although our findings suggest that moving from unemployment to part-time and full-time employment was associated with increases in mental health, it is possible that the association is partly due to reverse causation. Fixed effects models examine employment status and mental health at the same point in time, therefore it is not possible to form definitive conclusions about the direction of effect (Gunasekara et al., 2014). While there is strong evidence from previous research that changing employment status is causally related to changing mental health (Kuhn et al., 2009; Paul & Moser, 2009; Pierce & Schott, 2020), there is also evidence that declines in mental health led to changes to employment status (Olesen et al., 2013; Schuring et al., 2007). Secondly, it is possible that some important time varying factors such as household income, bereavement, area level employment shocks were not included in regression models. Missing data on variables and survey non-response is a common problem in longitudinal studies and may lead to selection bias. There is a large proportion of missing data for some variables in the HILDA survey, approximately 20% for the MHI-5 score and 16% for working hours. Loss to follow-up is another major limitation of this study, with only 40.8% of people in the sample participating in all 5 waves; and the response rate for new participants varied between 62% and 67% at each wave (Department of Social Service, 2022a, Department of Social Services, 2022b), which may have led to selection bias, potentially underestimating the magnitude of the effects given that people with health conditions are more likely to drop out of the survey, have poorer mental health, and be unemployed.

All variables in the study were self-reported. As such, there is potential measurement error in reporting disability status, employment status and mental health outcomes. It is possible that differential measurement error may occur when unemployed people over-report their disability and mental health to rationalize their economic inactivity (Black et al., 2017). However, the MHI-5 scale is an effective and well-validated measure with high specificity and sensitivity (Yamazaki et al., 2005), which is likely to minimize measurement error in mental health. The definition of disability group we used may have addressed potential misreporting of disability in a single wave of HILDA. Employment status is less likely to be subject to misclassification due to the nature of the question which specified people's employment status in the previous week. Finally, as the HILDA study participants with higher socio-economic status were less likely to be survey non-respondents than those with lower socio-economic status, this may affect generalisability.

## 5 Conclusion

Part-time employment is positively associated with the mental health of people with disability, of similar magnitude to full-time employment, especially for people younger than 45 years. The likely mental health benefits of part-time employment have important implications for the health and wellbeing of people with disability, and are also likely to have wider impacts, such as reductions in the use of mental health services and reliance on income support payments, which may have benefits to the economy.

## Funding

The work was supported by the 10.13039/501100000925National Health and Medical Research Council (1116385) and WISE Employment (N/A).

## Disclaimer

The findings and views reported in this paper are those of the authors and should not be attributed to either DSS or the Melbourne Institute.

## Ethics approval

The author asserts that all procedures contributing to this work comply with the ethical standards of the relevant national and institutional committees on human experimentation and with the Helsinki Declaration of 1075, as revised in 2008. Approval was granted from the Department of Social Services to the author for general access to HILDA and the primary data collection for HILDA has existing comprehensive ethics coverage.

## Data sharing

The data used are available free of charge to researchers through the Australian Government Department of Social Services (DSS). Access is available to users registered with the DSS Longitudinal Studies Dataverse and ADA Dataverse and is conditional on signing the Confidential Deed Poll.

## Ethical statement

All procedures contributing to this work comply with the ethical standards of the relevant national and institutional committees on human experimentation and with the Helsinki Declaration of 1075, as revised in 2008. The HILDA Survey was approved by the University of Melbourne Human Ethics Committee (ID 1955879).

## Author statement

**Lu Ye**: Methodology, Analysis, Writing - Original Draft, Anne Marie Kavanagh: Conceptualization, Methodology, Writing – Review & Editing, Supervision.

Dennis Petrie: Conceptualization, Writing – Review & Editing, Helen Dickinson: Conceptualization, Writing – Review & Editing, Funding acquisition.

Zoe Aitken: Conceptualization, Methodology, Writing – Review & Editing, Supervision.

## Declaration of competing interest

We declare no competing interests.

## Data Availability

The authors do not have permission to share data.
