# Supplementary material for: Part-time versus full-time employment and mental health for people with and without disability
Source: SSM Popul Health. 2023 Jun 7;23:101446. doi: 10.1016/j.ssmph.2023.101446 (PMC10275711; doi:10.1016/j.ssmph.2023.101446)
Supplement: Multimedia component 1 [file mmc1.docx]

# **­­­****Appendix A. Supplementary Table**

# Supplementary Table 1. Sample characteristics measured at each person’s first wave of data, by employment status

|  | Unemployed  n=1,721 (%) | Employed part-time  n=5,522 (%) | Employed full-time  n=8,739 (%) |
| --- | --- | --- | --- |
| Age (%) | | |  |
| 15-24 years | 876 (51.3) | 2,055 (39.6) | 1,339 (15.6) |
| 25-34 years | 356 (20.9) | 914 (17.6) | 2,529 (29.5) |
| 35-44 years | 211 (12.4) | 800 (15.4) | 1,730 (20.2) |
| 45-54 years | 165 (9.7) | 775 (14.9) | 1,858 (21.7) |
| 55-64 years | 99 (5.8) | 650 (12.5) | 1,109 (13.0) |
| Sex (%) | | |  |
| Male | 865 (50.3) | 1,735 (31.4) | 5,453 (62.4) |
| Female | 856 (49.7) | 3,787 (65.6) | 3,286 (37.6) |
| Country of birth (%) | | |  |
| Australia | 1,460 (84.9) | 4,608 (83.5) | 6,964 (79.7) |
| Other English Speaking | 91 (5.3) | 397 (7.2) | 791 (9.1) |
| Non-English speaking | 169 (9.8) | 516 (9.4) | 982 (11.2) |
| Household structure (%) | | |  |
| Couple no children | 237 (15.5) | 1,186 (22.9) | 2,798 (34.2) |
| Couple with children | 757 (49.5) | 2,790 (54.0) | 3,675 (44.9) |
| Single no children | 207 (13.5) | 484 (9.4) | 1,139 (13.9) |
| Single with children | 328 (21.5) | 710 (13.7) | 566 (6.9) |
| Disability status (%) | | |  |
| No | 1,060 (69.6) | 3,972 (80.2) | 6,837 (86.0) |
| Yes | 463 (30.4) | 980 (19.8) | 1,115 (14.0) |
| Education (%) | | |  |
| Bachelor’s degree or higher | 181 (10.5) | 1,314 (23.8) | 2,920 (33.4) |
| Dip or Cert. | 437 (25.4) | 1,484 (26.9) | 3,279 (37.5) |
| Secondary education | 330 (19.2) | 1,205 (21.8) | 1,273 (14.6) |
| Less than secondary education | 773 (44.9) | 1,517 (27.5) | 1,265 (14.5) |
| Parents occupation (%) | | |  |
| Never worked or low skill | 392 (23.7) | 743 (13.6) | 1,211 (14.1) |
| Medium skill | 599 (36.2) | 1,858 (34.1) | 2,889 (33.7) |
| High skill | 662 (40.1) | 2,846 (52.3) | 4,470 (52.2) |
| MHI-5 (mean±SD) | 64.7±20.0 | 72.6±17.4 | 74.9±16.0 |
